# How to let go: pectin and plant cell adhesion

**DOI:** 10.3389/fpls.2015.00523

**Published:** 2015-07-14

**Authors:** Firas Bou Daher, Siobhan A. Braybrook

**Affiliations:** The Sainsbury Laboratory, University of Cambridge, Cambridge, UK

**Keywords:** cell adhesion, pectin, polygalacturonase, pectin methylesterase, cell separation

## Abstract

Plant cells do not, in general, migrate. They maintain a fixed position relative to their neighbors, intimately linked through growth and differentiation. The mediator of this connection, the pectin-rich middle lamella, is deposited during cell division and maintained throughout the cell’s life to protect tissue integrity. The maintenance of adhesion requires cell wall modification and is dependent on the actin cytoskeleton. There are developmental processes that require cell separation, such as organ abscission, dehiscence, and ripening. In these instances, the pectin-rich middle lamella must be actively altered to allow cell separation, a process which also requires cell wall modification. In this review, we will focus on the role of pectin and its modification in cell adhesion and separation. Recent insights gained in pectin gel mechanics will be discussed in relation to existing knowledge of pectin chemistry as it relates to cell adhesion. As a whole, we hope to begin defining the physical mechanisms behind a cells’ ability to hang on, and how it lets go.

## Introduction

Most plant cells maintain a fixed position during development, attached to their neighbors by a shared cell wall interface. Since plant development relies on the harmonious combination of cell division, cell expansion and cell differentiation, it is essential that individual cells coordinate their development with that of their neighbors- with precise maintenance of cell adhesion or permission of cell separation when required. Within this review we will paint a picture of the interconnected roles of the cell wall and the cytoskeleton in cell adhesion, and in its release, by summarizing data from across decades and species.

In order to understand how the cell wall mediates cell–cell adhesion, we must first examine its composition and organization, focusing on the primary cell wall. Polysaccharides (mainly cellulose, hemi-cellulose, and pectin) represent about 90% of the cell wall mass with the remaining 10% comprising structural and polysaccharide-modifying proteins (Albersheim et al., 1996). Cell wall polysaccharides are synthesized at the level of the plasma membrane or delivered via the cytoskeleton and the secretory pathway (Moore and Staehelin, 1988; Lerouxel et al., 2006; Toyooka et al., 2009; Kang et al., 2011; Worden et al., 2012; Kim and Brandizzi, 2014). Modifying proteins are also delivered by cytoskeletal routes, and as such these materials and their delivery are key to understanding cell adhesion and separation. Furthermore, we must understand how the cell wall interface between two cells is formed, organized, and maintained.

In *Arabidopsis* leaves, roughly 50% of the cell wall is pectin and it comprises the matrix in which the cellulosic elements are embedded (Zablackis et al., 1995; Harholt et al., 2010). Pectin polysaccharides are galacturonic acid polymers and are represented by three major types: homogalacturonan (HG), rhamnogalacturonan-I (RG-I), and rhamnogalacturonan-II (RG-II) (Atmodjo et al., 2013). Pectic polysaccharides are synthesized in the golgi and delivered to the cell wall by secretory vesicles moving primarily along the actin cytoskeleton (Toyooka et al., 2009; Kim and Brandizzi, 2014), although there is recent evidence for kinesin-dependent pectin delivery via microtubules (Zhu et al., 2015).

**FIGURE 1:**
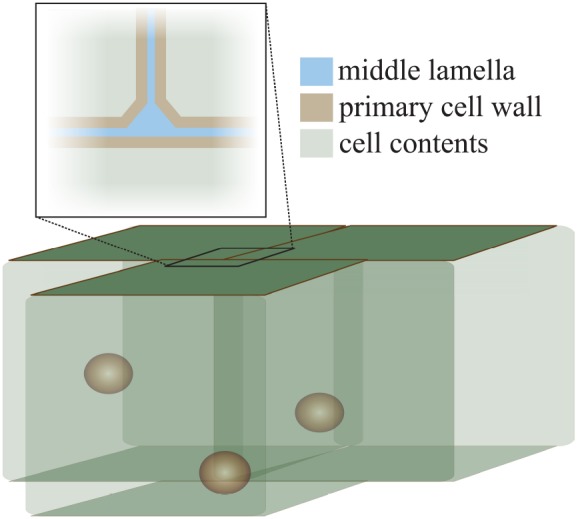
**The structure of the cell wall at the cell–cell interface.** This diagram illustrates the position of the middle lamella (pectin-rich, blue) and the primary cell walls (pectin-hemicellulose-cellulose, brown) at the junction of three cells. The characteristic “tri-junction” is evident. Spheres inside cells represent cell nuclei for illustration.

The cell wall is formed during cell division when a cell plate is formed between two new cells, resulting from a massive directed exocytosis, and possible contributions from endocytosis, of HG-pectin-containing vesicles (Dhonukshe et al., 2006; Reichardt et al., 2007; Miart et al., 2014; Drakakaki, 2015). Soon afterward, cellulose synthases arrive, hemicellulose delivery commences, and a new wall is generated for each cell with a pectin-rich area, the middle lamella, between them (Figure 1). Callose is also deposited at the cell plate during cytokinesis, but after cell division ends it is restricted to the plasmodesmata in the primary walls of growing cells (Northcote et al., 1989; Scherp et al., 2001). As such, the pectin-rich middle lamella is the major physical mediator of cell adhesion and separation. For the bulk of this review we will focus on the role of pectin, and its modifiers, in the middle lamella, and on their role in maintaining cell adhesion or permitting cell separation.

## Holding on: The Establishment and Maintenance of Cell Adhesion

The middle lamella between two cells is rich in pectin; its levels and chemical modification are key to regulating adhesion. Modification of pectin affects its ability to gel and act as glue between cells. HG pectin is gelled by calcium-mediated crosslinking. Newly delivered HG-pectin is highly methyl-esterified which makes it more fluid. The activity of a wall-modifying protein, pectin methyl-esterase (PME), removes the methyl groups of HG. De-esterified HG is readily cross-linked by calcium leading to a stiffer material and altering the mechanical properties of the cell wall (Micheli, 2001; Willats et al., 2001; Peaucelle et al., 2011; Braybrook et al., 2012). PME activity can be counteracted by the activity of another family of cell wall proteins, pectin methyl-esterase inhibitors (PMEIs) and as such the balance of these two proteins and their activities have effects on the mechanical properties of the middle lamella.

**FIGURE 2:**
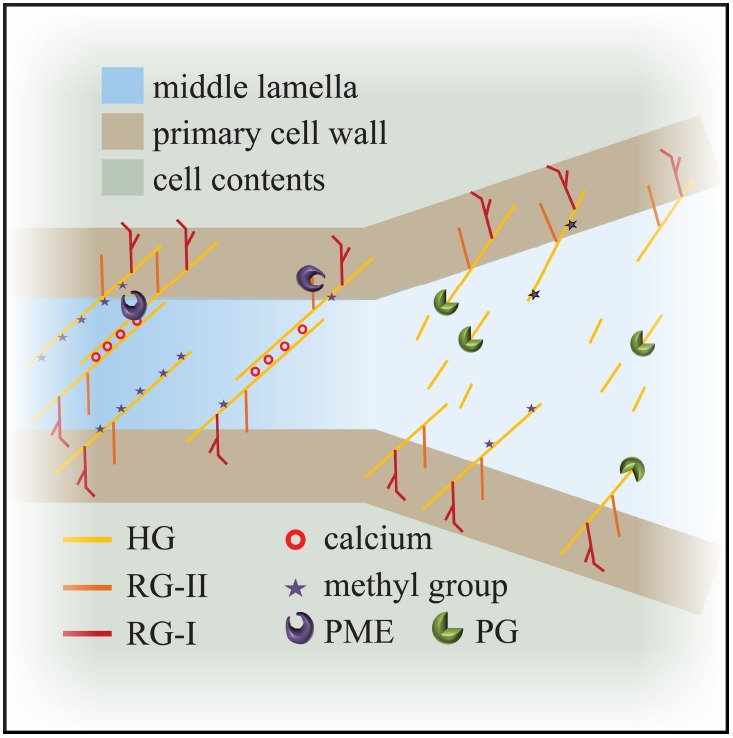
**Model for cell adhesion and cell separation.** Cross linking of the de-esterified pectin polymers maintains cell adhesion at the level of the middle lamella. Degradation of the de-esterified pectins by enzymes like polygalacturonases weakens connections and leads to cell separation. HG: homogalacturonan; RG: rhamanogalacturonan; PME: pectin methyl-esterase; PG: polygalacturonase.

Homogalacturonan pectin, in its de-esterified or low esterified form, is found in the middle lamella and in the corners of cell junctions (Figure 2; Bush et al., 2001; Parker et al., 2001; McCartney and Knox, 2002; Guillemin et al., 2005). Since de-esterified HG tends to form Ca^2+^ gels readily it is also important to note that calcium ions are enriched in the middle lamella (Figure 2; Rihouey et al., 1995; Huxham et al., 1999; Bush et al., 2001). The role of HG-Ca^2+^ gels in cell adhesion is underscored by the effects of treatment with calcium chelators such as EDTA (ethylenediaminetetraacetic acid), HMP (sodium hexametaphosphate) and CDTA (1,2-Diaminocyclohexanetetraacetic) which result in cell separation in various plants (Letham, 1960; Ng et al., 2000; McCartney and Knox, 2002). *Arabidopsis pme3* mutants, as well as lines overexpressing the PME inhibitors AtPMEI-1 and AtPMEI-2, display an increased efficiency in protoplast isolation from leaf mesophyll tissue, which indicates that cells were less adhesive and more easily separated from each other (Lionetti et al., 2015).

The effect of PME alteration is not *Arabidopsis* specific, implying a wide role for PMEs in cell adhesion across species; anti-sense-mediated down-regulation of PME in tomato fruit led to a loss of fruit integrity and a change in the ionic composition of the fruit (Tieman and Handa, 1994). The importance of pectin in adhesion even extends beyond land plants; the calcium cross-linked HG-rich extracellular matrix of the green algae *Penium margaritaceum* has been shown to be crucial for cell adhesion (Domozych et al., 2014). Together, these data position pectin de-esterification and calcium-mediated gelling as a key positive regulator of cell wall adhesion.

Given the role of HG methyl-esterification in pectin gelling, it follows that the methyl transferases which act during pectin biogenesis are key for adhesion as well. Localized in the golgi, they transfer methyl groups onto newly synthesized HG-pectin. Mutations in putative methyl-transferases in *Arabidopsis* have severe effects on growth and cell adhesion. The *qua2* mutant shows a 50% reduction in HG and severe cell adhesion defects (Mouille et al., 2007). The *qua2*-allelic *tumorous shoot development* (*tsd2*) mutant shows cell adhesion defects in the shoot apex, leaves and hypocotyl (Frank et al., 2002; Krupková et al., 2007). Interestingly, neither *qua2* nor *tsd2* show a difference from wild-type in their relative pectin esterification levels, evidence which indicates that while relative amounts of esterification may not be important absolute levels may be. Additionally, mutants in, or over expression of, the closely related methyl-transferase *QUA3* show no change in cell adhesion (Miao et al., 2011), indicating that the roles of different methyl-transferases in cell adhesion are likely highly specific, or alternatively highly redundant, in a *QUA* family containing 29 genes in *Arabidopsis* (Atmodjo et al., 2013).

Supporting a hypothesis for a pectin-level-effect on cell adhesion (and resultant effect on the de-methyl-esterified pectin level), several glycosyl transferase mutants display cell adhesion defects; glycosyl transferases are responsible for pectin synthesis in the golgi. The *quasimodo-1* (*qua1*) mutant in *Arabidopsis* displays reduced HG content, a decreased esterification level and cell adhesion problems (Bouton et al., 2002; Leboeuf et al., 2005). Note that *qua1* is also defective in xylan biosynthesis (Orfila et al., 2005). The *ectopically parting cells 1* (*epc1*) mutant affecting a glycosyl transferase displays reduced cell adhesion in the cotyledons and hypocotyl (Singh et al., 2005). When these data are taken into account it becomes clear that while the balance between esterified and de-esterified pectin is important, so is the overall level of HG pectin.

As previously introduced, there are two other pectins to consider as well—RG-I and RG-II, although their roles in cell adhesion are more complex and less well studied. In tobacco, the *nolac-H18* mutant has reduced RG-II pectin and exhibits crumbled shoots and abnormal meristem cell adhesion indicating a role in adhesion (Iwai et al., 2002). On the other hand, in the *Arabidopsis echidna* (*ech*) mutant RG-I and xyloglucan are low but cell adhesion is wild-type (Gendre et al., 2011, 2013). These discrepancies indicate specificities in pectin-mediated cell adhesion that extend beyond a simple story where pectin-equals-glue. They hint at a complex story for pectin within the middle lamella and its influences on cell adhesion.

## Keeping it Together: Actin and Cell Adhesion

The delivery of pectin and its modifying proteins occurs mainly via the actin cytoskeleton. It is therefore unsurprising that defects in actin filament organization affect cell adhesion. The Actin-related protein2/3 complex (Arp2/3) is highly conserved and is the key component in regulating branching and nucleation of actin filaments (Higgs and Pollard, 2001). Mutants in ARP2/3 complex subunits have been characterized in *Arabidopsis* where they are associated with disorganization of the actin cytoskeleton, defects in cell shape, and ectopic cell separation in hypocotyls (Le et al., 2003; Li et al., 2003; Mathur et al., 2003a,b; El-Assal et al., 2004; Saedler et al., 2004). Mutants in up-stream regulators of the Arp2/3 complex also display defects in cell adhesion as seen in the *spike1* mutant (Qiu et al., 2002). Interestingly, no difference in cell wall composition between wild-type and the *arp2* mutant has been observed. The only observed difference was an abnormal thickening at the three-way wall junction of the mutant, possibly indicating altered composition at the middle lamella (Dyachok et al., 2008). We still have only a basic understanding of how actin structure might ultimately affect cell adhesion, and we cannot exclude effects on wall components beyond pectin; but the evidence presented here points toward the delivery of components and pectin-modifying proteins to the cell wall.

It is perhaps not just delivery of components and modifying proteins to the cell wall that affect adhesion but also their recycling. Actin is a key player in endocytosis in plants, yeast and animals (Moreau et al., 1997; Roszak and Rambour, 1997; Schaerer-Brodbeck and Riezman, 2000; Insall et al., 2001; Merrifield et al., 2004; Benesch et al., 2005; Kaksonen et al., 2006). Given the adhesion defects described above, when actin is disrupted, it is plausible that actin-mediated endocytosis might also be involved in maintaining cell wall integrity. Recycling of cell wall components has been demonstrated in germinating *Arabidopsis* seeds and maize root tip cells (Baluška et al., 2002; Pagnussat et al., 2012). Cell wall modifying proteins may also be recycled, as seen in the case of PMEI endocytosis in growing pollen tubes (Röckel et al., 2008). These data suggest that recycling from the cell wall by endocytosis may be necessary to maintain cell wall integrity and cell adhesion, but this area needs to be further explored.

## Letting go: Cell Separation as a Necessary Developmental Process

During some developmental processes cell adhesion is purposefully dissolved leading to cell separation. For example, natural phenomena that require cell separation are observed in leaf abscission, fruit dehiscence, fruit ripening, tetraspore separation, pollen release and root cap cell sloughing. The study of these processes gives us an insight into the mechanisms controlling cell adhesion and separation in plants. Next we will examine how pectin (and its regulation) contributes to the phenomenon of cell separation.

Unsurprisingly, given its role in adhesion, there are several examples of pectin alterations which block regulated cell separation. Inhibition of PME activity prevents separation of root border cells in pea (Wen et al., 1999). In *Arabidopsis*, the mutants *quartet1* (*qrt1*), a PME, and *quartet3* (*qrt3*), a polygalacturonase (PG), result in the failure of tetraspores separation (Rhee et al., 2003; Francis et al., 2006). This implies that both PME and PG activity are necessary to separate the tetrads: PME removes the methyl groups from HG and subsequently PG breaks down the pectins, releasing the individual pollen grains (Figure 2). With respect to cell separation, it is worth considering the interplay between PME and PG in some more detail.

Polygalacturonases are enzymes that cleave de-esterified HG backbones via hydrolysis; as such they depend on PME activity. They are represented by a large gene family in *Arabidopsis* with diverse expression profiles (Kim et al., 2006; González-Carranza et al., 2007). PGs have been implicated as positive regulators of cell separation, fruit ripening, abscission, cell growth and dehiscence (Jenkins et al., 1999; Sander et al., 2001; Atkinson et al., 2002, 2012; González-Carranza et al., 2007; Xiao et al., 2014). Mutations in the PG coding genes *QRT2* and *QRT3* lead to problems in organ dehiscence, abscission and tetraspore separation in *Arabidopsis* (Rhee et al., 2003; Ogawa et al., 2009). PGs are involved in silique dehiscence in *Arabidopsis* and *Brassica* (Jenkins et al., 1999; Roberts and McCann, 2000; Sander et al., 2001) and silique development is also accompanied by an increase in PME activity which reinforces the interconnected roles of PME and PG (Louvet et al., 2011). These analyses indicate that PG-mediated, PME-dependent, pectin degradation is a key event in cell separation during development.

Our information surrounding the role of PG in promoting cell separation goes well beyond *Arabidopsis*, again highlighting an ancient role for pectin in cell connectivity: overexpression of a PG1 subunit, *OsBURP16*, in rice decreased cell adhesion and overexpression of PG in apple caused premature leaf shedding due to reduced adhesion in the abscission zone (Atkinson et al., 2012; Liu et al., 2014). Conversely, down-regulation of PG in apples increased fruit firmness and cell adhesion (Atkinson et al., 2012). This correlates well with findings in strawberry where the down-regulation of PG reduced fruit softening (Quesada et al., 2009). Interestingly, the effect of PG alteration in tomato is incongruent with all other evidence. Down-regulation of a fruit-ripening-specific PG in tomato only slightly reduced fruit softening (Kramer et al., 1992; Langley et al., 1994). In line with this phenotype, the down-regulation of PG only yielded a slight reduction in pectin de-polymerisation in fruits (Brummell and Labavitch, 1997). Lastly, overexpression of PG in tomato could restore ripening in a ripening and softening inhibited mutant (*rin*) but not softening (Giovannoni et al., 1989). While these data indicate that PG has only a minor role in tomato fruit ripening (in contrast to strawberry and apple, as above), PG activity was much higher in tomato fruit homogenates compared to the intact tissue indicating that PG mediated softening in tomato may be regulated less by the quantity of the enzyme, and more by activity through the biochemical environment (Kramer et al., 1992). Overall, there is a strong trend for the importance of PG in mediating cell separation, further underlining the role of pectin in the process as well.

## Contradictions that Highlight Complexity

Throughout this review, we have seen several instances of contradictory evidence surrounding the role of pectin in cell adhesion and separation. For example, in one tissue PME activity promotes adhesion and in another separation: high esterification level reduces cell adhesion in the mesophyll and the pericarp (Tieman and Handa, 1994; Lionetti et al., 2015), but simultaneously causes increased cell adhesion and blocks cell separation in tetraspores and root border cells (Wen et al., 1999; Rhee et al., 2003). It is likely that this difference is due to a complex mix of other modifying proteins and a complex biochemical environment; as an illustration, the presence of PG in ripening fruit would increase the likelihood that de-esterified pectin would be depolymerised, not cross-linked with Ca^2+^. This does not negate the importance of pectin and the middle lamella, but instead highlights the complexity of cell adhesion and separation.

The activity of PMEs is also highly diverse. The *Arabidopsis* genome contains 66 *PME*-related genes (Tian et al., 2006) and what little we know about their activity indicates they are highly regulated. Solution pH has been shown to affect the activity of PMEs in persimmon and apple (Alonso et al., 1997; Denès et al., 2000) and PME activity is also salt dependent (reviewed in, Jolie et al., 2010). To make the situation more complex, it is important to recall that PME activity can be counteracted by PMEI proteins, and interestingly some of the predicted PMEs also contain inhibitor domains (Tian et al., 2006). We still have very little information on how most of the PMEs are specifically regulated and very little idea about their developmental specificity. Again, we have more evidence that adhesion and separation are complex processes worthy of dissection.

## Additional Components in the Mix

While the middle lamella is mostly pectin, it also contains some hemicellulose. As such, it is not unexpected that xyloglucans have been implicated in fruit softening (Rose and Bennett, 1999; Vicente et al., 2007). Immuno-labelling of hemicellulose (LM15 antibody) in unripe fruits of tomato showed signal in the wall at points of cell adhesion, which was lost in ripe fruit. As in the case of pectins, it is not only altered levels of hemicellulose that affect cell adhesion, but also the modification of existing hemicellulose and its effect on cell wall structure as a whole. The wall loosening protein expansin modifies the connection between hemicellulose and cellulose; *EXPANSIN1* (*Expa1*) is involved in tomato fruit ripening and its down-regulation reduced the amount of pectin de-polymerisation (Brummell et al., 1999). This data simply reinforces the complex nature of the cell wall and cell adhesion.

## Summary

In the end, we can make some well-founded conclusions about the role of pectin in cell adhesion and separation. The physical position of the middle lamella, its pectin-rich nature and its accumulation of calcium all point to a crucial role for pectin in these processes. As with any cell wall-mediated process the effect of transgenic and mutational analyses is complicated by redundancy and compensation, and so our current understanding is limited. Experimental evidence also tells us that the tissue specific context involving other modifying proteins, their deposition and recycling and the biochemical environment are also critical. In spite of these difficulties, it is clear that pectin and calcium are required for proper cell adhesion and that pectin modification and degradation are strictly required for cell separation in its various developmental contexts. The details of which enzyme performs which task in which tissue, how altering delivery of such modifying proteins or pectin itself might regulate connectivity, the role of other wall components and the cytoskeleton remain to be ironed out. With current advances in experimental techniques and interests in adhesion and separation growing, understanding these processes is an achievable and exciting goal.

### Conflict of Interest Statement

The authors declare that the research was conducted in the absence of any commercial or financial relationships that could be construed as a potential conflict of interest.
